# Toward aitiopoietic cognition: bridging the evolutionary divide between biological and machine-learned causal systems

**DOI:** 10.3389/fcogn.2025.1618381

**Published:** 2025-07-17

**Authors:** Tomas Veloz

**Affiliations:** Departamento de Matemáticas, Universidad Tecnológica Metropolitana, Santiago, Chile

**Keywords:** artificial intelligence, emergence, causal reasoning, autopoieisis, metasystem transitions, embodied cognition, synthetic biology

## Abstract

We examine and compare autopoietic systems (biological organisms) and machine learning systems (MLSs) highlighting crucial differences in how causal reasoning emerges and operates. Despite superficial functional similarities in behavior and cognitive abilities, we identify profound structural differences in how causality is operationalized, physically embodied, and epistemologically grounded. In autopoietic systems, causal reasoning is intrinsically tied to self-maintenance processes across multiple organizational levels, with goals emerging from survival imperatives. In contrast, MLSs implement causality through statistical optimization with externally imposed objectives, lacking the material self-reorganization that drives biological causal advancement. We introduce the concept of “aitiopoietic cognition”—from Greek “aitia” (cause) and “poiesis” (creation)—as a framework where causal understanding emerges directly from a system's self-constituting processes. Through analyzing convergence pathways including evolutionary algorithms, material intelligence, homeostatic regulation, and multi-scale integration, we propose a research program aimed at bridging this evolutionary divide. Such integration could lead to artificial systems with genuine intrinsic goals and materially grounded causal understanding, potentially transforming our approach to artificial intelligence and deepening our comprehension of biological cognition.

## 1 Introduction

Machine learning systems (MLSs) are rapidly increasing their influence in our lives, transforming sectors from healthcare to entertainment (Marcus and Davis, 2019; LeCun et al., 2015), and where substantial investments are flowing into their sophistication (Maslej et al., 2025), there is an increasing need to understand the fundamental differences between “us and them” (Bengio et al., 2024). For clarity and analytical precision, we will refer to “them” as MLSs, acknowledging their primary mechanism of development and adaptation.

Considering the perspective of a child first learning to distinguish entities in a computer-interface level, or for an alien visiting our planet, there is no major difference between humans and MLSs. Both can read, understand text and images, type and draw, speak, and engage in a stream of complex actions including planning abilities, communication skills, and even abstract reasoning about concepts, causal relationships, and reflexive understanding of self and others. This similarity is recognized as well in robotic interfaces that allow for physical interaction and movement (Moro et al., 2019; Manzi et al., 2020). This seemingly remarkable similarity is often explained through the lens of computational functionalism (Putnam, 1967; Chalmers, 1996), which posits that mental states are defined by their functional roles rather than their physical substrate. Under this view, if MLSs functionally replicate human cognitive processes, even from a completely different substrate, they can be considered fundamentally equivalent—and hence subjected to comparison at the agential level (Goertzel, 2007).

### 1.1 The “equivalence hypothesis” for testing causal cognition in human and machines

By looking more closely at how we compare ourselves and machines, we arrive at the strong influence that has played the Turing test, which is assumed to be known by the reader. While it aims at testing thinking, it more precisely tests the ability to engage in a conversation using previously learned information, and hence it does not test thinking directly, but learning and the ability to express such learning (Moor, 1976). In this article we do not want to dig into the definitions of learning, thinking and intelligence and how that impacts our understanding of artificial intelligence (see Wang, 2019 for such analysis). Instead, we want to explore the consequences of comparing the causal cognitive abilities of a machine and a human using input-output architectures. Mainstream cognitive science implicitly assumes that these architectures “mean the same” for both humans and machines. In fact, the input-output architecture is, as well, generally believed to be responsible for the learning process, in an equivalent way, for both humans and MLSs. The latter is accepted and well-justified by a large number of successful scientific programs in a variety of fields including various branches of psychology and cognitive science (Anderson, 2007), linguistics (Fodor, 1975; Chomsky, 1986; Pinker, 1994), computer science (Russell and Norvig, 2020), ethology (Lorenz, 1981), and others (Clark, 2001). These have shown that the design of processes and experiments based on input-output architectures have a high inductive power and allow to explain how learning, adaptation, and the development of increasingly complex responses (behaviors) to environmental challenges can be generated/stopped or enhanced/inhibited. Therefore, it is assumed that the way in which the “processing unit” that transforms input to output, i.e. the “black-box” for MLSs or “the mind” for humans, has no particular difference for what concerns defining cognition (Chalmers, 1996; Clark and Chalmers, 1998).

However, the above implicit equivalence assumption masks profound differences in how the inner workings of the material implementations of the input and output, and more crucially of them together with the “processing unit” forming a full system, shapes the existential, developmental and evolutionary features of cognition in MLSs and humans (Deacon, 2011).

At the existential level, cognition in Humans and other biological organisms is implemented within autopoietic systems—self-creating and self-maintaining entities that constantly regenerate their components through metabolic processes that harness energy from its environment (Boden, 1999; Maturana and Varela, 2012; Thompson, 2007). In contrast, cognition in MLSs is implemented in physically static machines whose embodiment remains unchanged. At the developmental level autopoietic systems develop through multi-level structures based on cells made of molecular networks (Fields and Levin, 2022; Witkowski et al., 2023), each level having its own sense of self and its own competences resembling cognitive abilities aligned to their particular physical instantiation (which might or might not implement universal Turing computation), while for MLSs their development is based on external assemblage without multi-level structures and no internalized sense of self, and a “single level of intelligence” evolves through algorithmic adjustments and data-driven feedback in a universal Turing machine setting.

At the evolutionary level, these differences amplify what cognition means at each substrate. Autopoietic systems have evolved through natural selection operating on genetic variations across billions of years, with multiple major transitions creating hierarchical levels of organization (Smith and Szathmáry, 1995; Szathmáry, 2015). This evolutionary process has resulted in systems where purposeful behavior emerges from the intricate interplay between material constraints and informational dynamics at multiple scales (West et al., 2015; Heylighen, 2023). In stark contrast, MLSs “evolve” through directed human engineering, following developmental trajectories on a fixed Von Neumann architecture, optimized for specific). purposes involving performance, economic cost, size, and computation speed, rather than survival in open-ended environments (Stanley and Lehman, 2015). Their evolutionary trajectory lacks the self-organized complexity and emergent properties characteristic of biological evolution, instead following design principles imposed externally by human developers with predetermined objectives (Lake et al., 2017).

### 1.2 Goals as a criteria for comparing causal cognition

Instead of focusing on performance to test causal cognition, we can focus on “goal-directedness,” as goals reflect the source of actions in the processing unit (Heylighen, 2023). Following this idea, goals serve as a crucial pivot point for comparing the causal cognition between humans and machines (Deacon, 2011). In autopoietic systems, goals are defined by the relation between the imperative for the system's physical existence through self-preservation and the ways available to interact with its environment (Kolchinsky and Wolpert, 2018). For MLSs, goals are externally defined optimization targets disconnected from any necessity of material self-preservation. This difference in the origin and nature of goals highlights an important gap in how their causal cognition operates. This aspect raises issues regarding the comparison of other significant aspects of cognition such as intelligence and adaptation (see Stano et al., 2023, and other articles in that special issue).

This paper examines the fundamental characteristics of autopoiesis and machine learning, analyzes their key differences using goals as a reference concept that concern causal cognition, and explores potential convergence in futuristic systems that integrate their diverse “cognitive scaffoldings” (Ziemke et al., 2004). Finally we outline a path toward bridging this evolutionary divide (Witkowski et al., 2023; Seth, 2021), by proposing “aitiopoietic cognition”—from Greek “aitia” (cause) and “poiesis” (creation)—as a framework where causal understanding emerges directly from a system's self-constituting processes, creating a recursive relationship between physical organization and causal reasoning.

## 2 Autopoietic systems: from survival to goals

Autopoietic systems, introduced by biologists Humberto Maturana and Francisco Varela in the 1970s, are defined as networks of processes that produce the components necessary for their continued existence and boundary maintenance. This concept provides a cybernetic-inspired framework for understanding biological autonomy (Maturana and Varela, 2012). Unlike mechanistic or vitalistic accounts of life, autopoiesis offers a naturalistic perspective that emphasizes the dynamic, process-oriented nature of living systems (Weber and Varela, 2002).

### 2.1 The inner-outer structure

From a biochemical perspective, autopoietic systems operate through metabolic networks that continuously transform matter and energy to regenerate their components while maintaining organizational stability. This self-production occurs through thermodynamically open processes that sustain the system far from equilibrium (Moreno and Mossio, 2015). The continuous production of a semi-permeable boundary distinguishes the system from its environment while regulating internal processes and external exchanges, creating a fundamental inside-outside asymmetry crucial for biological autonomy (Luisi, 2003).

A defining characteristic of autopoietic systems lies in their hierarchical organization across spatial and temporal scales. This multi-level architecture enables nested autonomy: molecular networks maintain metabolic closure, cells exhibit decision-making via signaling pathways (Gao et al., 2023), and tissues coordinate via biophysical feedback (Forgacs and Newman, 2005). Each level sustains operational closure while contributing to higher-order autopoiesis, exemplifying Varela's concept of “autonomous identity at several levels” (Varela, 1979). This organization aligns with Salthe's (1985) hierarchical evolution framework and enables bidirectional causality: “downward causation” (Ellis, 2012), where higher levels modulate lower-level processes, and “upward constraints,” where molecular dynamics limit higher-level possibilities (Kauffman, 1993; West-Eberhard, 2003).

The operational closure of autopoietic systems does not preclude dynamic engagement with environments; rather, it enables multi-level structural coupling—a process by which recurrent interactions trigger compensatory changes while preserving organizational coherence (Di Paolo, 2005; Moreno and Mossio, 2015). This coupling operates hierarchically: molecular networks couple with intracellular conditions, cells with tissue microenvironments, and organisms with ecological niches, each level maintaining its autonomy while contributing to the system's viability (Maturana and Varela, 2012; Salthe, 1985). Evolutionary pressures sculpt this hierarchy, favoring modularity for robustness (Wagner, 1996) and degeneracy (Edelman and Gally, 2001) to buffer against perturbations.

Crucially, coupling is asymmetrical and structurally determined: the environment does not dictate changes but perturbs the system, whose architecture—shaped by evolutionary and developmental history—filters which perturbations are salient (Barandiaran and Moreno, 2008; Juarrero, 1999). A cell's membrane receptors selectively respond to extracellular ligands while its metabolic state constrains receptor expression, illustrating how multi-level dependencies mediate environmental interactions (West-Eberhard, 2003; Huang, 2012; Rafelski and Theriot, 2024).

Thus, autopoietic systems enact their worlds through multi-scalar, history-laden interactions—a process where autonomy and dependency coexist, and every perturbation becomes an opportunity for meaning-making (Varela et al., 1991; Barandiaran et al., 2009).

### 2.2 Autopoietic cognition

The connection between autopoiesis and cognition emerges from the system's need to maintain itself through adaptive interactions with its environment. As Maturana and Varela provocatively stated, “living is knowing,” suggesting that even basic autopoietic systems exhibit a primitive form of cognition through their selective environmental coupling (Thompson, 2007). This perspective reframes cognition not as information processing but as sense-making—transforming neutral environmental stimuli into meaningful distinctions relevant to continued existence (Di Paolo and Thompson, 2014).

The transition of autopoietic systems into sense-making adaptive agents hinges on their capacity to develop multi-level regulatory hierarchies that monitor and modulate viability conditions across scales (Di Paolo, 2005; Moreno and Mossio, 2015). From these processes goal-directedness becomes naturally linked to autopoietic organization. Autopoietic systems exhibit “purposive” behavior because their structure—forged through structural coupling (Juarrero, 1999)—embodies historical solutions to viability challenges (Deacon, 2011). For instance, slime molds optimize nutrient networks via self-organizing gradients (Nakagaki et al., 2000). This naturalized teleology (Weber and Varela, 2002) proposes a solution to the paradox of purpose as entities in the future influencing its past: goals are emergent properties of systems that recursively couple action to self-maintenance across scales (Barandiaran et al., 2009).

The formal representation of goals in autopoietic systems presents unique modeling challenges precisely because goals emerge from the system's organization rather than being explicitly encoded (Veloz, 2021). Several mathematical frameworks have been developed to capture this emergence, each highlighting different aspects of how purpose arises from process.

Dynamical systems theory provides the broadest framework, representing autopoietic goals as attractor states in state space that maintain viability amid perturbations (Heylighen, 2023; Kauffman, 1993). Crucially, these attractors shift based on the system's internal state, creating a landscape where goals are context-dependent rather than fixed. This adaptive landscape model has been formalized in work on viability boundaries and adaptive control (Barandiaran and Egbert, 2014), providing mathematical tools to analyze how autopoietic systems generate and modify goals in response to changing conditions.

More specific mathematical frameworks address different aspects of goal-directedness. Chemical Organization Theory (Dittrich and Speroni di Fenizio, 2007; Veloz and Razeto-Barry, 2017) models self-maintaining chemical networks where closure and self-production create stability conditions that serve as implicit goals. This approach enables rigorous analysis of how chemistry creates persistent identity—a precondition for purpose—by identifying organizational closure in reaction networks. Meanwhile, Free Energy Principle models (Friston, 2010; Priorelli et al., 2025) formalize how predictive regulation serves autopoietic maintenance, representing goals as probability distributions over viable states that systems act to maintain through active inference. This Bayesian approach provides a computational bridge between autopoietic goals and machine learning frameworks. Agent-based modeling has become increasingly important for modeling how goals emerge from simple behavioral mechanisms tied to viability constraints, demonstrating how selection pressures can drive the emergence of increasingly complex goal hierarchies through simulated evolution (Froese and Ziemke, 2009; Packard et al., 2019).

While the conceptualization of goals is thoroughly integrated with theoretical frameworks of autopoiesis, and the relationship between goal-directedness and biological purpose is well-established in philosophical terms, the formal mathematical representation of these concepts remains in its infancy. Current models capture aspects of emergent purpose but struggle to represent the full richness of biological goal-directedness—particularly the multi-scale competencies that comprise biological intelligence (Witkowski et al., 2023; Fields and Levin, 2022). These competencies, from basal cognition in single cells to the abstract reasoning of complex organisms, suggest multiple forms of intelligence operating across different scales and materialities, challenging our current modeling capabilities. As autopoietic theory continues to evolve, bridging the gap between rich theoretical accounts and precise formal representations remains a crucial frontier—one that will not only deepen our understanding of biological cognition but also provide insights for developing artificial systems with more naturalistic forms of goal-directedness (Veloz, 2021; Thompson, 2007; Di Paolo, 2005).

### 2.3 From autopoiesis to aitiopoiesis

The transition from autopoietic to aitiopoietic cognition, i.e., going from achieve material self-preservation to embody affordances through behaviors that resemble causal reasoning, represents a fundamental leap where systems transcend mere organizational closure to actively constitute causal knowledge through their very existence (Deacon, 2011). This transition becomes evident when examining how autopoietic systems generate what we term “agential causality”—causal understanding that emerges not from abstract computation but from the material processes of self-constitution and environmental coupling. Consider bacterial chemotaxis: the cell's sensory-motor apparatus doesn't simply detect gradients but constitutes a knowledge-generating system where the phosphorylation cascade dynamics simultaneously maintain cellular organization and create understanding about environmental cause-effect relationships (Davies and Levin, 2023; Levin, 2019). The bacteria's tumble-and-run behavior emerges from constitutional processes where causal learning and self-maintenance are inseparably intertwined—the system literally embodies its causal models through the recursive dynamics of its own material organization.

Recent advances in synthetic multicellularity illuminate this transition by revealing how collective systems can exhibit emergent aitiopoietic properties that exceed their individual components' autopoietic capabilities. Xenobots, constructed from amphibian skin and cardiac cells, demonstrate a primitive form of aitiopoietic cognition where collective behavior emerges from cellular self-organization without genetic programming or external control circuits (Moreno and Etxeberria, 2005; Newman and Bhat, 2009; Kriegman et al., 2020). These “living robots” navigate their environment through constitutive processes—their locomotion, object manipulation, and collective coordination arise from the same self-maintaining dynamics that preserve their multicellular integrity. Crucially, their behavioral competencies represent endogenous properties of agential materials rather than externally imposed algorithms, suggesting that aitiopoietic cognition scales naturally from autopoietic foundations when appropriate organizational architectures emerge. Similarly, Anthrobots self-assemble from human lung cells into motile spheroids with cilia-driven propulsion and tissue-repair capabilities, demonstrating how multicellular collectives can exhibit goal-directed behaviors that emerge from, rather than being programmed into, their constitutional dynamics (Solé et al., 2024).

The synthetic biology framework reveals aitiopoiesis as fundamentally involving multi-scale agency where causal competencies emerge through hierarchical coupling between different levels of organization (Solé et al., 2016). In organoid systems, individual cells contribute to tissue-level morphodynamic reasoning—the collective navigation of anatomical morphospace through perception-action loops that simultaneously maintain tissue architecture and generate knowledge about spatial relationships (Solé et al., 2024). This represents a form of “collective aitiopoietic cognition” where causal understanding emerges from the constitutional dynamics of cellular collectives, not from pre-programmed instructions. The tissue “learns” about its environment through the very processes that constitute its existence—growth gradients, mechanical forces, and bioelectrical patterns become both the medium of self-maintenance and the substrate of causal reasoning. Importantly, this scaling of aitiopoietic competency reveals a crucial principle: higher-order causal understanding doesn't reduce to lower-level mechanisms but emerges through what Levin terms “agential materials”—substrates with intrinsic competencies that can be guided through behavioral interventions rather than mechanical control.

The implications for artificial aitiopoietic systems are profound. Current synthetic approaches reveal that genuine aitiopoiesis cannot be engineered through traditional top-down design but must emerge from substrates that exhibit “competency in transcriptional, anatomical, and physiological problem spaces” (Davies and Levin, 2023). The failure of purely computational approaches to achieve constitutional causality suggests that future aitiopoietic systems will require physical substrates where information processing affordances such as pattern recognition and causal reasoning as well as self-maintenaning affordances such as reparation and duplication are materially unified rather than functionally separated (Gill et al., 2025). This points toward a research program focused not on programming artificial agents but on cultivating synthetic systems where aitiopoietic cognition can emerge from the recursive dynamics of embodied self-organization, potentially through hybrid bio-synthetic architectures that combine the constitutional properties of living materials with the scalability of artificial substrates.

## 3 Machine learning systems: from goals to causality

Machine learning represents the current paradigm to artificial intelligence by allowing algorithms to improve through experience by learning from data (Russell and Norvig, 2020). Early machine learning focused on representational approaches and decision trees, but the field underwent a transformation with the advent of deep learning architectures that could automatically extract hierarchical features from raw data (LeCun et al., 2015). This evolution reflects a broader transition from engineering-centric to data-centric approaches, where system behavior emerges from statistical patterns rather than explicit design.

### 3.1 Machine learning instantiations of causal cognition

At its core, machine learning operates through statistical inference and optimization processes that adjust internal parameters to minimize prediction errors or maximize reward signals. MLSs operationalize causality through statistical associations rather than mechanistic or teleological reasoning. Rooted in pattern recognition, these systems optimize for predictive accuracy by minimizing loss functions (e.g., cross-entropy, mean squared error) that measure deviations from training data distributions (Goodfellow et al., 2016). According to Pearl's (2019) Causal Hierarchy, ML systems operates at Level 1 (observational inference), lacking capacity for intervention (Level 2) or counterfactual reasoning (Level 3). For instance, deep neural networks trained on medical datasets may correlate hospital beds with patient mortality without inferring beds as sites of treatment rather than causation (Geirhos et al., 2020). Such spurious correlations stem from ML's reliance on statistical shortcuts—surface features that maximize training accuracy but fail to capture causal invariance (Arjovsky et al., 2019).

This process can be formalized mathematically as finding a function f^*^ such that prioritizes empirical risk minimization:


(1) $$\begin{array}{l} f^{*}=argmin_{f}E\left[L\left(f\left(x\right),y\right)\right], \end{array}$$

where *E* means expected value, *L* quantifies prediction error over a dataset *D*. While effective for interpolating training distributions, this formulation conflates correlation with causation, as models lack mechanisms to distinguish confounding variables (Schölkopf, 2022). For example, ML systems trained on socioeconomic data often reproduce biased associations (e.g., race and loan default rates) due to dataset imbalances rather than causal relationships (Koh et al., 2021).

Recent critiques highlight how this statistical foundation limits ML's causal robustness. Adversarial attacks—minor input perturbations that deceive models (Szegedy et al., 2013)—expose the fragility of associational reasoning, while distribution shifts (e.g., hospital data from urban vs. rural settings) degrade performance catastrophically (Koh et al., 2021). Unlike biological systems, which evolved to prioritize causally salient features (e.g., predators, nutrients), ML lacks evolutionary pressure to distinguish signal from noise, rendering its causal cognition inherently shallow (Marcus and Davis, 2020).

### 3.2 Engineered causal architectures and the limits of extrinsic goals

Contemporary machine learning (ML) systems attempt to integrate causal reasoning through architectures that blend statistical learning with formal causal frameworks. Structural causal models (SCMs) represent one approach, applying Pearl's do-calculus (Pearl, 2019) to infer interventions from observational data. Tools like DoWhy (Sharma and Kiciman, 2020) and CausalNex based on Bayesian DAGs (Zheng et al., 2018) operationalize this by encoding causal graphs, yet they require human-specified variables and struggle with latent confounders—a critical limitation in real-world datasets (Schölkopf et al., 2021). For example, in healthcare, SCMs often fail to account for unmeasured socioeconomic factors that mediate treatment outcomes (Kaddour et al., 2022).

Causal reinforcement learning (CRL) extends this by training systems to learn intervention policies. DeepMind's Causal Meta-RL (Ke et al., 2022) demonstrates how systems can infer task structure through trial-and-error interventions, yet their objectives remain static (e.g., maximizing game scores). Unlike biological systems, which dynamically repurpose goals (e.g., switching from foraging to predator evasion), CRL systems lack mechanisms to reconfigure objectives in response to existential needs (Lake et al., 2017).

Neuro-symbolic hybrids merge neural networks with symbolic logic to enforce causal rules (Sheth et al., 2023). However, these rules are externally imposed rather than emergent from self-constitution, rendering them brittle under novel scenarios where “goal-directed commonsense is needed” (Garcez and Lamb, 2023). For instance, symbolic constraints in autonomous driving systems (e.g., “stop at red lights”) fail to adapt when road conditions defy predefined norms (e.g., emergency vehicles). Table 1 summarizes the limitations of causality from our goal-oriented perspective.

**Table 1:** Training method for achieving goals and limitation examples.

**Training paradigm**	**Causal limitation**	**Example**	**References**
Supervised (accuracy)	Fails under distribution shift	Medical diagnosis	Geirhos et al., 2020
Reinforcement (reward)	No adaptive repurposing of objectives	Game score maximization	Ke et al., 2022
Neuro-symbolic (rules)	Brittle to novel scenarios needing commonsense	Autonomous driving	Garcez and Lamb, 2023

These architectures reveal a fundamental limitation regarding their goals: MLS goals are extrinsic optimizations (e.g., loss minimization), and hence they decouple causal reasoning from variables that underlie the actions their knowledge shall engage into Marcus and Davis (2020).

## 4 Key causal reasoning differences between autopoietic systems and machine learning systems

We now organize the differences between how Autopoietic Systems and MLS relate to causal reasoning into key dimensions that help to identify further venues for their scientific study.

### 4.1 Operationalization of goals and causality

Causality in autopoietic systems is fundamentally recursive and self-referential, rooted in their operational closure. These systems' fundamental goal is to remain alive, i.e., maintain their identity through circular cause-effect chains that simultaneously produce and depend on their own boundaries (Maturana and Varela, 2012). For instance, a cell's metabolic network synthesizes the very components—enzymes, membranes, and organelles—that enable its continued existence. Here, causality is inseparable from the system's teleological imperative: self-preservation. Perturbations to autopoietic systems (e.g., nutrient deprivation) trigger adaptive responses aimed at restoring homeostasis, illustrating how causal reasoning is intrinsically directed toward sustaining systemic coherence (Luisi, 2003). The system's “goal” is not external but emergent of its self-reinforcing organizational structure.

In contrast, causality in machine learning (ML) systems is extrinsically defined by statistical correlations derived from training data. ML models, such as deep neural networks, infer patterns through gradient-driven optimization, with no inherent representation of counterfactuals or physical mechanisms (Pearl, 2019). For example, a convolutional neural network trained to classify images associates pixel configurations with labels (e.g., “cat” or “dog”), but these associations lack intrinsic grounding in the system's structure. The “goal” of an ML system—minimizing a loss function—is imposed externally by designers, reflecting no existential imperative. While autopoietic systems exhibit endogenous causality (causal processes emerge from self-maintenance), ML systems rely on exogenous causality, where causal attribution is bounded by the scope and biases of training datasets (Marcus, 2018) or rules imposed (Marcus and Davis, 2020). We summarize our discussion in Table 2.

**Table 2:** Summary of operational differences between autopoietic and machine learning causal reasoning systems.

**Dimension**	**Autopoietic systems**	**Machine learning systems**
Causal mechanism	Recursive, based on self-referential loops at multiple hierarchical levels	Non-recursive, based on statistical correlations and gradient optimization
Teleological basis	Endogenous (self-maintenance)	Exogenous (loss minimization)
Boundary definition	Operational closure (self-produced membrane/organization)	Data distribution and algorithmic constraints

### 4.2 Material embodiment and causal reasoning improvement

In autopoietic systems, causal reasoning improves through physical embodiment of increasingly complex goals. The material substrate available by its current operational organization, under the right mutations, directly enables the development of sophistication through what Heylighen (1995) calls meta-system transitions. These transitions enable new levels of control, i.e., the ability to handle perturbations in novel ways. This process is physically instantiated—cellular differentiation creates novel causal potentials through material reorganization that enables emergent functions. Major evolutionary transitions (Szathmáry, 2015) demonstrate how material reorganization drives causal advancement. When independent autopoietic units integrate into higher-order collectives, they physically restructure to enable a larger entity with new causal capabilities not only in relation to its environment but also with respect to its own components via top-down control (Rosas et al., 2020). This physical restructuring creates multi-level regulatory networks where causality operates across nested spatial and temporal scales—from molecular recognition (microseconds) to memory formation (decades).

This material integration enables the expansion of goal-directed structures across wider spatial domains and longer temporal horizons, termed the “care cone” (Witkowski et al., 2023). Long-term planning in mammals emerges not from computational scaling but from physical evolution of neural structures that materially integrate multiple internal states. In contrast, MLSs develop causal reasoning through pathways decoupled from their material embodiment. Their physical substrate remains static while abstract parameters adjust, creating a thermodynamic disconnect between energy expenditure and causal improvement. Neural networks expend identical energy regardless of whether they're refining causal models or reinforcing spurious correlations (Thompson et al., 2020).

This thermodynamic decoupling constrains how causal reasoning improves in MLSs. Without material reorganization that extends control across scales, causal advancement becomes purely instrumental—serving externally defined objectives without extending capacity for self-maintenance, or any other existential notion embedded in it. MLS causal improvements depend primarily on data quantity rather than material reorganization. While evidence suggests resemblances of major evolutionary transitions, by the identification of phase-transition-like improvements when crossing certain network size thresholds (Schölkopf et al., 2021), these remain qualitatively different from evolutionary transitions. ML systems expand within predefined architectural constraints without generating novel material configurations that enable fundamentally new forms of causal reasoning.

The latter explains why biological causal reasoning exhibits open-ended improvement while artificial causality remains bounded by its instrumental nature and thermodynamic decoupling. We summarize our discussion in Table 3.

**Table 3:** Differences on how autopoietic and ML systems improve their causal cognition.

**Aspect**	**Machine learning systems**	**Autopoietic systems**
Mechanism of Improvement	Parameter optimization within fixed architecture	Material reorganization across multiple scales
Thermodynamic Relationship	Energy use unrelated to causal advancement	Energy expenditure aligned with control capabilities
Temporal integration	Reconstruction through increasingly smaller processing timescales	Nested timescales from molecular to evolutionary
Evolutionary potential	Bounded by architectural constraints	Open-ended through major transitions
Self-reorganization	No physical restructuring during learning	Continuous physical adaptation to maintain viability

### 4.3 The attribution of causality

The epistemological foundations of causal attribution differ radically between autopoietic and machine learning systems, illuminating a philosophical dimension that combines their divergent material and operational principles.

#### 4.3.1 Mechanistic causality in autopoietic systems

In autopoietic systems, causal attribution is mechanistic and grounded in material interactions. The experimental methods used to understand biological causality—such as knockout studies in yeast that reveal causal roles of genes in glycolysis (e.g., *PGI1* deletion halts glucose metabolism)—directly manipulate physical components to observe system-wide effects (Kitano, 2002), and can alter or be altered by features at other scales through mechanisms that might involve for example electrical, chemical and fluid-dynamics levels (Levin, 2019). The epistemology of causation is tied to physically observable and manipulable processes, such as enzyme-substrate binding observed via crystallography or electron microscopy.

This mechanistic approach to causality reveals the ontic nature of autopoietic causation—causal relationships are embedded in the system's physical structure and processes rather than being observer-dependent constructs. When biologists identify a gene as causal for a phenotype, they are identifying material pathways through which physical interactions propagate effects (Bechtel and Richardson, 2010). The multi-level organization of these systems means that causality operates simultaneously across molecular, cellular, and organismal scales, with bidirectional influences between levels.

#### 4.3.2 Instrumental causality in machine learning systems

In contrast, causal attribution in ML systems is instrumental and *post hoc*. Researchers employ techniques such as ablation studies (removing a neural network layer) or Shapley values (Lundberg and Lee, 2017) to approximate feature importance, but these approaches provide statistical approximations rather than revealing physical causal mechanisms. When an ML researcher identifies a feature as “causal,” they are making an epistemic claim about statistical relationships rather than identifying a physical pathway.

The epistemology of ML causality remains fundamentally statistical—a high Shapley value for a pixel in an image classification task doesn't imply a physical causal pathway but instead indicates a statistical correlation that the model has learned to exploit. This distinction becomes critical when deploying ML systems in real-world contexts where causal understanding (rather than correlation) is necessary for safe and effective operation.

## 5 Complementarity through evolutionary perspectives

Despite the fundamental differences between autopoietic systems and MLS outlined in previous sections, several promising pathways for complementarity are emerging. These approaches address the key gaps we've identified—particularly in goal formation, material embodiment, and causal understanding—offering potential bridges across this evolutionary divide.

### 5.1 Evolutionary algorithms and open-ended exploration

The application of evolutionary principles to machine learning design represents a significant step toward more biologically-inspired systems. Unlike traditional optimization approaches that focus on maximizing predefined metrics, evolutionary algorithms emphasize diversity, adaptation, and the emergence of novel solutions (Lehman and Stanley, 2011). Recent advances in quality diversity algorithms and Multi-dimensional Archive of Phenotypic Elites (MAP-Elites) demonstrate how maintaining behavioral diversity rather than focusing solely on performance leads to more robust and creative solutions that better resemble biological adaptability (Mouret and Clune, 2015; Cully et al., 2015).

Open-ended evolution research extends this approach by creating computational environments where continuous innovation emerges without predefined fitness functions (Taylor et al., 2016). These systems begin to address the goal-directedness gap identified in Section 4.1 by enabling the discovery of novel objectives rather than merely optimizing predefined ones. As Packard et al. (2019) note, truly open-ended artificial systems require mechanisms that support not just optimization but the continuous emergence of new ways to define and pursue goals—a property inherent to biological evolution (Boden, 1998).

However, these approaches still operate primarily at the algorithmic level, with limited impact on the material substrate gap highlighted in Section 4.2. The challenge remains integrating open-ended exploration with physical embodiment, a fundamental requisite for aitiopoietic cognitive systems.

### 5.2 Embodied cognition and material intelligence

A more direct approach to bridging the material gap involves developing artificial systems where materiality plays a constitutive role in cognition. Beyond conventional robotics, which often implements disembodied algorithms in physical shells, emerging research explores how material properties themselves can perform computational functions (Pfeifer et al., 2007; Paul, 2006).

Recent work in morphological computation demonstrates how physical dynamics can replace explicit computation, allowing systems to leverage material properties for intelligent behavior (Hauser et al., 2011; Müller and Hoffmann, 2017). Soft robotics and programmable materials enable adaptive behavior through their intrinsic material properties rather than through explicit programming (Laschi and Cianchetti, 2014; Rieffel et al., 2009). These approaches begin to address the thermodynamic decoupling identified in Section 4.2 by creating systems where physical structure directly participates in information processing.

The challenge remains developing materials that not only compute but also maintain and regenerate themselves—a defining aspect of autopoietic systems. However, the “aitiopoietic” integration of autopoietic structures into causal and other forms of reasoning is still in its infancy (McMillen and Levin, 2024).

### 5.3 Homeostatic regulation and predictive processing

The active inference framework (Friston, 2010; Friston et al., 2017) offers a computational approach that potentially bridges autopoietic self-maintenance and machine learning. By framing cognition as the minimization of surprise (or free energy) through continuous prediction and updating, this framework provides a computational account of how systems maintain homeostasis while adapting to environmental challenges.

Recent implementations in artificial systems demonstrate how predictive architectures can develop intrinsic goals related to maintaining viable states (Baltieri and Buckley, 2019). Particularly promising is research integrating homeostatic regulation directly into neural network architectures. Lechner et al. (2021) have demonstrated neural networks with homeostatic mechanisms that maintain internal stability while adapting to external challenges, exhibiting a primitive form of the intrinsic goal-directedness characteristic of autopoietic systems.

These approaches begin to address the causality gap identified in Section 4.3 by grounding causal understanding in the system's own viability conditions rather than in purely statistical correlations. However, they still operate primarily within the computational domain, with limited connection to physical self-maintenance. In this vein, conceptual advancement has been made by Kolchinsky and Wolpert (2018) by defining the concept of “semantic information” that refers to the Shannon information responsible for its viability, i.e., its self-production in autopoietic terms. However, this measure has not been yet properly linked to self-production but to proxy viability formulas that are informational as well.

### 5.4 Multi-scale integration and hierarchical agency

The hierarchical nature of autopoietic systems—with their nested levels of organization and regulation—finds a parallel in emerging approaches to multi-scale artificial intelligence. Recent advances in hierarchical reinforcement learning and multi-agent systems demonstrate how collective intelligence can emerge from interactions between simpler agents operating at different scales (Domingo-Fernández et al., 2022).

When designed with appropriate structural coupling between levels, these systems can develop emergent goals and coordination patterns reminiscent of biological collectives (Levin et al., 2023). This multi-scale approach potentially addresses the causal attribution gap identified in Section 4.3 by enabling both bottom-up and top-down causation across different levels of organization. This is consistent with evolutionary proposals that focus not only on individual but group selection and cooperation mechanisms, that have been proven useful in biology and culture (Wilson, 1975; Foster et al., 2017; Wilson et al., 2023), and being recently adopted as an alternative foundational paradigm in economics (Wilson and Snower, 2024).

## 6 Discussion

We examined the fundamental differences between autopoietic systems (biological organisms) and machine learning systems (MLSs), focusing on how their different natures affect causal cognition. We proposed that goals serve as a crucial pivot point for comparing these systems and explored potential convergence paths toward autopoietic systems that can integrate with MLS to embody “aitiopoietic cognition.”

Our analysis proceeded by first establishing the apparent functional similarity but fundamental structural difference between humans and MLSs cognition. We then examined autopoietic systems in depth, highlighting their self-organizing nature, emergent goals, and multi-level organization. Next, we analyzed how MLSs implement causal reasoning through statistical inference and optimization with externally imposed goals. This comparative framework allowed us to identify key differences in causal reasoning between these systems across three crucial dimensions: the operationalization of goals and causality, material embodiment and improvement mechanisms, and the epistemological foundations of causal attribution. Table 4 summarizes these fundamental operational, substrate, and epistemological differences, providing a concrete framework for understanding the evolutionary divide between biological and machine-learned causal systems.

**Table 4:** Comparative analysis of autopoietic systems and machine learning systems across operational, substrate, and epistemological dimensions.

**Dimension**	**Autopoietic systems**	**Machine learning systems**
Goal formation (operational)	Emergent from closure and self-maintenance imperatives	Externally imposed by designers
Causality implementation (operational)	Feedback-driven: within internal state via structural coupling	Statistical correlations and pattern recognition in data distributions
Physical embodiment of information (substrate)	Material substrate dynamically encode-decode information achieving computation through autopoietic processes	Static mapping between a specific physical part in charge of information states and other independent part in charge processing
Improvement process (substrate)	Physical reorganization across multiple scales enables enhanced control capabilities	Abstract parameter adjustment while maintaining fixed material architecture
Energy-cognition coupling (substrate)	Thermodynamic processes directly linked to cognitive enhancement and self-maintenance	Thermodynamic disconnect between energy expenditure and causal improvement
Causal knowledge source (epistemological)	Constitutional causality arising from material self-organization and boundary maintenance	Statistical inference from training data patterns and correlational structures
Causal attribution (epistemological)	Grounded in existential imperatives and viability conditions of the system itself	Based on computational optimization of externally defined loss functions
Learning foundation (epistemological)	Sense-making through autonomous environmental coupling and meaning generation	Information processing through supervised/unsupervised pattern extraction

The differences illustrated in Table 4 reveal profound implications for how we understand and develop artificial intelligence. Autopoietic systems exhibit recursive, self-referential causality rooted in their operational closure, with goals emergent from self-maintenance imperatives. Their causal reasoning improves through physical reorganization enabling multi-scale control, with material embodiment directly participating in cognition. In contrast, MLSs operate through extrinsic optimization and statistical correlations, with their physical substrate remaining static while abstract parameters adjust—creating a thermodynamic disconnect between energy expenditure and causal improvement.

Our analysis suggest the need for integrating both cognitive architectures, including ML and autopoietic and perhaps others, with philosophical questions about causality and agency. Recent striking examples, reviewed in section “From Autopoiesis to Aitiopoiesis,” demonstrate how these philosophical concepts find concrete expression in biological examples. Xenobots and organoids illustrate how collective behavior can emerge from cellular self-organization without external programming, suggesting that aitiopoietic properties can scale naturally from autopoietic foundations when appropriate organizational architectures emerge (Kriegman et al., 2020; Davies and Levin, 2023).

Therefore, the aitiopoietic cognition research program should proceed through four complementary tracks: (1) minimal aitiopoietic systems—engineering simple chemical/cellular systems that exhibit constitutional causality using synthetic biology techniques. For example, sensitivity and robustness of specific components might unveil causal-like behavior (Shinar et al., 2009); (2) measurement frameworks for characterizing aitiopoiesis—operationalizing goal-directedness by compatibilizing matter and information processing interplays. The latter requires an integration of notions from dynamical systems theory related to autopoiesis such as homeostatic recovery times, attractor basin and viability analysis, with information theoretical metrics explaining how the information of a system is processed in relation to its existence as a collective (Friston, 2010; Kolchinsky and Wolpert, 2018; Rosas et al., 2020); (3) scaling constitutional cognition—understanding how aitiopoietic properties emerge in a collective through multi-scale modeling approaches (Szathmáry, 2015); and (4) Hybrid Bio-Synthetic architectures—combining living materials with artificial substrates to achieve increasingly complex goal-directed behavior (Witkowski et al., 2023; Fields and Levin, 2022).

However, current convergence pathways face significant limitations: evolutionary algorithms encounter computational intractability in open-ended settings, material intelligence lacks genuine self-maintenance capabilities, homeostatic approaches remain primarily computational rather than constitutional, and multi-scale integration struggles with embodiment challenges. These represent current research frontiers rather than insurmountable barriers, suggesting that breakthrough progress will require novel approaches that transcend these individual pathway limitations. These convergence challenges are summarized in Table 5.

**Table 5:** Convergence pathways between autopoietic and ML systems.

**Convergence pathway**	**Addresses key gap**	**Current limitations**	**Exemplar research**
Evolutionary algorithms	Goal formation	Limited material impact	MAP-Elites (Mouret and Clune, 2015)
Material intelligence	Physical embodiment	Lacks self-maintenance	Soft robotics (Laschi and Cianchetti, 2014)
Homeostatic regulation	Causal grounding	Primarily computational	Neural homeostasis (Lechner et al., 2021)
Multi-scale integration	Causal attribution	Limited embodiment	Multi-agent systems (Levin et al., 2023)

## 7 Conclusion

The future of artificial intelligence may lie in systems that are neither fully machine nor fully living, but that bridge this evolutionary divide through novel forms of embodied, self-maintaining cognition. From here, we suggest that a research program aiming to reach “aitiopoietic cognition” should be developed. This ambitious research program convergence pathway involves the development of synthetic systems that exhibit genuine autopoietic properties—self-creation, self-maintenance, and self-boundary definition within their simulation domains. Research in synthetic biology and artificial life aims to create minimal chemical systems that display these properties (Luisi, 2003; Stano et al., 2023).

The realization of this research program will require studying metasystem transitions (Heylighen, 1995) and major evolutionary transitions (Szathmáry, 2015) in open-ended evolutionary settings. Metasystem transitions represent moments when systems develop new levels of control and coordination, fundamentally transforming their problem spaces (Witkowski et al., 2023) and enabling novel forms of causal reasoning. By creating artificial environments where such transitions can emerge naturally, we may navigate problem spaces with increasingly sophisticated “care-cones” (Witkowski et al., 2023)—expanding the spatial and temporal horizons over which they can exercise meaningful causal influence based on intrinsic rather than externally imposed goals (Deacon, 2011). This expansion of the care-cone would represent a crucial step toward artificial systems that can adapt to complex, open-ended environments through genuine understanding rather than mere statistical optimization (Seth, 2021). As Fields and Levin (2022) have argued, such systems would demonstrate competence across multiple domains through intrinsically motivated exploration and materially grounded causal reasoning (Walsh, 2015), potentially transforming our understanding of both biological and artificial intelligence.

This ambitious synthesis suggests a new understanding of cognition and agency that might bring us closer to resolving the mind-body problem, not through theoretical abstraction, but through the concrete development of systems that embody both material self-maintenance and sophisticated causal understanding.

The development of artificial entities with genuine intrinsic goals and materially grounded causal understanding raises fundamental ethical considerations that must be systematically addressed within this research program. To maintain analytical rigor within current scientific understanding, this discussion focuses on early-stage developmental issues rather than interesting but more speculative scenarios, which have been explored elsewhere (Kurzweil, 2005; Aerts and Arguëlles, 2022; Vidal, 2024). The proposed research program emphasizes minimal systems with constrained operational scope, necessitating the establishment of comprehensive safety protocols for self-modifying systems, ensuring beneficial alignment of early aitiopoietic prototypes that prioritize incremental progress with systematic monitoring of emergent capabilities.

A particularly significant ethical consideration involves understanding the fundamental material-informational dynamics underlying experiential states in general systems—an investigation that must remain central throughout the development of this research program (Witkowski et al., 2023). Given that aitiopoietic systems represent partially autonomous entities with potential experiential capacities, ethical protocols require continuous revision in accordance with evolving scientific understanding of agency, sentience, and consciousness, in artificial systems. This iterative ethical framework becomes essential precisely because the research program's object of study may possess forms of experience or proto-sentience that demand careful moral consideration as these systems develop greater autonomy and complexity.

This ambitious synthesis suggests a new understanding of cognition and agency that might bring us closer to resolving the mind-body problem, not through theoretical abstraction, but through the concrete development of systems that embody both material self-maintenance and sophisticated causal understanding. By developing artificial entities with truly intrinsic goals and materially grounded causal understanding, we may finally bridge the evolutionary divide between life and machine while maintaining commitment to careful, ethically-guided progress.
